# Long memory and changepoint models: a spectral classification procedure

**DOI:** 10.1007/s11222-017-9731-0

**Published:** 2017-02-13

**Authors:** Ben Norwood, Rebecca Killick

**Affiliations:** Department of Mathematics and Statistics, Lancaster University, LA1 4YF Lancaster, UK

**Keywords:** Classification, Long memory, Changepoint, Wavelet spectrum, Non-stationarity

## Abstract

Time series within fields such as finance and economics are often modelled using long memory processes. Alternative studies on the same data can suggest that series may actually contain a ‘changepoint’ (a point within the time series where the data generating process has changed). These models have been shown to have elements of similarity, such as within their spectrum. Without prior knowledge this leads to an ambiguity between these two models, meaning it is difficult to assess which model is most appropriate. We demonstrate that considering this problem in a time-varying environment using the time-varying spectrum removes this ambiguity. Using the wavelet spectrum, we then use a classification approach to determine the most appropriate model (long memory or changepoint). Simulation results are presented across a number of models followed by an application to stock cross-correlations and US inflation. The results indicate that the proposed classification outperforms an existing hypothesis testing approach on a number of models and performs comparatively across others.

## Introduction

It is not often the case that a given data set has a known explicit model from which it is generated. Analysts will look to fit an appropriate model to such a series in the hopes of understanding the underlying mechanisms or to make predictions into the future. The models proposed are expected to be distinct in their properties such that there is a clear prevalence of a suitable model for the data. However, models with certain structural features have been known to have similar properties to other models (Granger and Hyung 2004). This overlap will be here referred to as an ‘ambiguity’ between the models. This is such that either model may appear similar to one another in some metrics, but provide very different interpretations on the data generating process, and lead to different predictions into the future.

In this paper, we consider the ambiguity between long memory and changepoint models. This ambiguity has been documented in fields such as finance and economics which are modelled using long memory models (Granger and Ding 1996; Pivetta and Reis 2007) and changepoint models (Levin and Piger 2004; Starica and Granger 2005). Thus, it is reasonable to assert that there is an element of ambiguity between these two models. Following the discussion and in-depth analysis within Diebold and Inoue (2001), it has been shown that both models share some similar properties, especially within the spectrum. Often a decision on a model cannot be made with the ‘luxury’ of prior knowledge, and as such assuming the data derives from either of these models comes at a risk of mis-specification.

Existing work in Yau and Davis (2012) conducts a hypothesis test to determine between the changepoint and long memory model. The authors choose to use the changepoint model as a null model with the justification that this is the more plausible model. However, in some circumstances this may not be the case, so it leads to the question as to which model should be the null model. It would be entirely feasible to choose the changepoint model as the null model, not reject $H_0$ and then flip to have the long memory model as the null model and also not reject $H_0$. This does not give a clear answer to the question of an appropriate model.

As an alternative this paper introduces a classifier, which places no such assumptions on which model is preferred. Instead, the purpose of a classifier is only to give a measure of which category provides the best fit. In the context here, it can measure which model best describes a time series, without assuming that this model is where the data were originally generated from. Classification of time series has been previously used in Grabocka et al. (2012) and Krzemieniewska et al. (2014). It was shown in Yau and Davis (2012) that the autocorrelation function and periodogram of data generated from a changepoint model and a long memory model exhibit similar structures (i.e. slow decay in the autocorrelation and spectral pole at zero). However, if we consider a time-varying periodogram, then the stationarity of a long memory model can be seen (constant structure over time), whilst a changepoint model exhibits the piecewise stationarity expected [see for example Killick et al. (2013)]. As the time-varying spectrum shows evidence of a difference between these models, we use it as the basis for our classification procedure.

The structure of this article is as follows. The background and methods to our approach are given in detail in Sect. 2. A simulation study of the proposed classification method, with a comparison to the likelihood ratio test from Yau and Davis (2012), can be found in Sect. 3. Applications of the classifier are then given using US price inflation and stock cross-correlations in Sect. 4. Finally, concluding remarks and a discussion is given in Sect. 5.

## Methods

### Changepoint and long memory models

The aim of our method is to distinguish between data which arise from either a changepoint or a long memory model. To define these, we first define the general autoregressive integrated moving average (ARIMA) model, characterised by its autoregressive (AR) parameters $\varvec{\phi } \in \mathbb {R}^p$, moving average (MA) parameters $\varvec{\theta } \in \mathbb {R}^q$ and the integration (I) parameter $d \in \mathbb {N}$. For random variables $X_1, X_2, \ldots , X_n$ this is formally defined as,

$$\begin{aligned} \left( 1 - \sum _{k=1}^p \phi _k B^k \right) \left( 1 - B \right) ^d X_t = \left( 1 + \sum _{k=1}^q \theta _k B^k \right) \epsilon _t \end{aligned}$$

where $\epsilon _t \sim N(0,\sigma ^2)$ and *B* is the backward shift operator such that $B X_t = X_{t-1}$ and $B \epsilon _t = \epsilon _{t-1}$. A variation of this, autoregressive fractional integrated moving average (ARFIMA), is such that $d \in \mathbb {R}$, allowing it to be fractional. This modification allows long memory behaviour to be captured through dependence over a large number of previous observations.

For the purpose of this paper, we define the changepoint and long memory models as:

1 $$\begin{aligned} X_t&\sim {\left\{ \begin{array}{ll} \mu _1 +\text {ARMA}(\varvec{\phi _1}, \varvec{\theta _1}) &{} \text {if} \quad t = 1,2, \ldots \tau \\ \mu _2+\text {ARMA}(\varvec{\phi _2}, \varvec{\theta _2}) &{} \text {if} \quad t = \tau + 1, \tau + 2, \ldots n.\\ \end{array}\right. } \end{aligned}$$

2 $$\begin{aligned} X_t&\sim \mu + \text {ARFIMA}(\varvec{\phi },d,\varvec{\theta }) \quad t = 1, 2, \ldots , n \end{aligned}$$

Note that we depict a single changepoint $\tau = \lfloor n\lambda \rfloor$ for notational ease, but the software we provide (see Sect. 5) contains the generalisation to multiple changes through use of the PELT algorithm (Killick et al. 2012) and extending Eq. (1) to include multiple $\tau$. Other models such as ARCH models and fractional Gaussian noise (Molz et al. 1997) could also be used, but we restrict our consideration to ARFIMA here. In the general case, we allow $p, q \in \mathbb {N}$, but in the simulations and applications given in Sects. 3 and 4 we restrict their range for computational reasons.

**Fig. 1:**
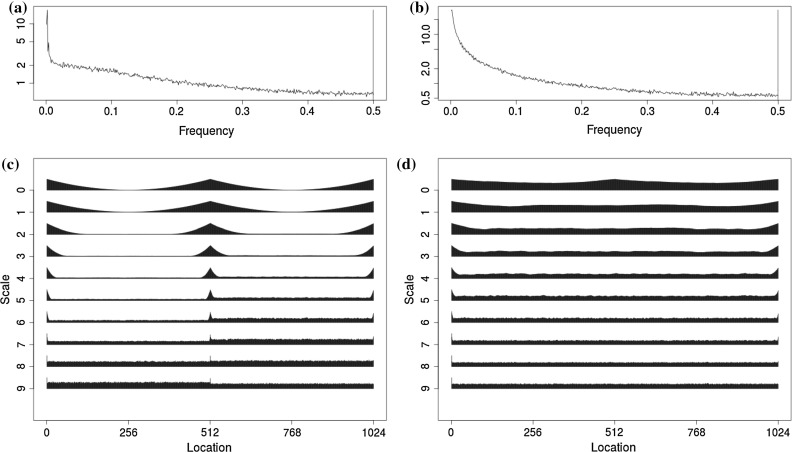
Empirical periodogram and wavelet spectrum averaged over 500 realizations. **a** Changepoint periodogram. **b** Long memory periodogram. **c** Changepoint wavelet spectrum. **d** Long memory wavelet spectrum

### Wavelet spectrum

The ambiguity present between diagnostics of the competing models given in Eqs. (1) and (2) can cause issues in identifying the correct model. Figure 1 shows the average empirical periodograms from realisations of long memory [ARFIMA(0, 0.4, 0)] and changepoint (AR(1), $\lambda =0.5$, $\phi _1=0.1$, $\phi _2=0.4$, $\mu _1=0$, $\mu _2=1$) models. It can be seen that the periodogram for the changepoint model has a pole at zero and shows similar behaviour to that of long memory.

Before discussing the wavelet spectrum, we provide a brief background to wavelets and the specific spectrum we propose to use.

Wavelets capture properties of the data through a location–scale decomposition using compactly supported oscillating functions. Through dilation and translation, a wavelet is applied across a number of a scales and locations to capture behaviour occurring over different parts of a series. Further information on them and their application can be found in Daubechies (1992) and Nason (2010). In this work, we use the model framework of the locally stationary wavelet process which provides a stochastic model for second-order structure using wavelets as building blocks.

We follow the definition in Fryzlewicz and Nason (2006) for a locally stationary wavelet (LSW) process.

#### Definition 1

Define the triangular stochastic array $\left\{ X_{t,N} \right\} ^{N-1}_{t=0}$ which is in the class of LSW processes given it has the mean-square representation

$$\begin{aligned} X_{t,N} = \sum _{j=1}^{\infty } \sum _{k} W_{j} \left( \frac{k}{n}\right) \psi _{j,k-t}\xi _{j,k}, \end{aligned}$$

where $j \in {1,2,\ldots }$ and $k\in \mathbb {Z}$ are scale and location parameters, respectively, $\psi _j = (\psi _{j,0}, \ldots , \psi _{j, L_{j} - 1})$ are discrete, compactly supported, real-valued non-decimated wavelet vectors of support length $L_{j}$. If the $\psi _j$ are Daubechies wavelets (Daubechies 1992) then $L_j = (2^j - 1)(N_h -1) + 1$ where $N_h$ is the length of the Daubechies wavelet filter, finally the $\xi _{j,k}$ are orthonormal, zero-mean, identically distributed random variables. The amplitudes $W_j (z) : [0,1] \rightarrow \mathbb {R}$ at each $j \ge 1$ are time-varying, real-valued, piecewise constant functions which have an unknown (but finite) amount of jumps. The constraints on $W_j (z)$ are such that if $\mathcal {P}_j$ are Lipschitz constants representing the total magnitude of jumps in $W^2_j (z)$, then the variability of $W_j (z)$ is controlled by

- $$\sum ^{\infty }_{j=1} 2^j \mathcal {P}_j < \infty $$,
- $$\sum ^{\infty }_{j=1} W_j^2 (z) < \infty $$ uniformly in *z*.

As in the traditional Fourier setting, the spectrum is the square of the amplitudes and as such the evolutionary wavelet spectrum can be defined as

$$\begin{aligned} S_{j} \left( \frac{k}{N} \right) = \left| W_j \left( \frac{k}{N} \right) \right| ^2 \end{aligned}$$

which changes over both scale (frequency band) *j* and location (time) *k*.

Considering both scale and location, the two dimensions allow the differences between the proposed models to be seen. Examples of the differences in these spectra are given in Fig. 1 for both the changepoint and long memory models. To interpret the wavelet spectrum: scale corresponds to frequency bands with high frequency at the bottom to low frequency at the top. Further details on the spectrum and its applicability can be found in Fryzlewicz and Nason (2006), Nason (2010) and Killick et al. (2013). Note that there is a clear difference between the wavelet spectra of the two models with the changepoint model being piecewise stationary (pre- and post-change), with the change occurring in the spectrum where the change occurs in the data. In contrast the long memory model remains flat across each scale and time reflecting the stationarity of the original series.

Due to the fact that the wavelet spectrum gives a distinction between the two models, we propose to use this as the basis for our inference regarding the most appropriate model. Whilst the Fourier spectrum could be used here as in Janacek et al. (2005), we choose to use the evolutionary wavelet spectrum. As shown in Fig. 1, this is advantageous for characterising the non-stationarity changepoint data due to the scale–location transformation used. This is since the $W_j (z)$ are constant for stationary models, but for non-stationary models the break in the second-order structure of the original data causes breaks in the wavelet spectra, as described in Cho and Fryzlewicz (2012).

In the next section, we detail how to use the wavelet spectrum of the two models in a classification procedure.

### Classification

Testing whether a long memory or changepoint model is more appropriate whilst under model uncertainty comes with the hazard of mis-specification. A formal hypothesis test places assumptions on the underlying model in both the null and alternative, but the allocation of the null is hazardous—should the changepoint model be the null or alternative? It would be entirely feasible to choose the changepoint model as the null model, not reject $H_0$ and then flip to have the long memory model as the null model and also not reject $H_0$. Given the absence of a clear null model, which result to proceed with is unclear. Instead, it may be preferable to quantify the evidence for each model separately. A classification method such as the one proposed here gives a candidate series a measure of distance from a number of groups, which can then be used to select the most appropriate group.

In the previous subsection, it was demonstrated that the wavelet spectrum can be used to distinguish the changepoint model from the long memory model, and the classifier proposed here builds on this. However, to begin a classification method must first ‘teach’ itself on the structure of the classes through sets of training data. These are data sets already determined to be in each category and are the basis for calculating the distances from each group. This previous knowledge allows for determination of patterns and features of each category (that are unique from other categories) for comparison to the candidate data set. A common example is the spam filter on mailboxes, which is trained on previous spam emails so that it can classify if a new email that arrives is spam or not. The decision is made by comparing it to a number of patterns already determined to be features in spam email for example, short messages or hidden sender identities. Further information on classification methods and training them can be found within Michie et al. (1994).

In our example, we only have a single data set of length *n*, the classifier has no previous information to train on. To remedy this we create training data through simulation. Given a candidate series we first fit the competing models in Eqs. (1) and (2) choosing the best fit for each model. For the changepoint model the best fit uses the ARMA likelihood within the PELT multiple changepoint framework to identify multiple changes in ARMA structure (Hyndman and Khandakar 2008; Killick et al. 2012). When considering fitted long memory models, a number of ARFIMA models are fitted (Veenstra 2012) and selection occurs according to Bayesian information criterion [following Beran et al. (1998)].

Following the identification of the best changepoint and long memory models, the training data are then simulated as (Monte Carlo) realisations from these, denoted by

$$\begin{aligned} \varvec{X}^g_m = \left\{ X^g_{i,m} \right\} _{i=1,2,\ldots ,n}&\quad m=1,2,\ldots ,M. \\&\quad g=1,2. \end{aligned}$$

where the group, $g = 1$ for changepoint simulations and $g=2$ for long memory simulations, *M* is the number of simulated series and *n* is the length of the original series. Note that we are not sampling from the original series, we are generating realizations from the fitted models.

Now we have the training data and the observed data, denoted $\varvec{X}^{o}$, a measure of distance of the observed data from each group is calculated. As discussed previously, we will use a comparison of their evolutionary wavelet spectra as the distance metric. Before detailing the metric, we first define the wavelet spectrum of the original series as

$$\begin{aligned} \varvec{S}^o = \left\{ S_{k}^o \right\} _{k = 1, 2, \ldots n*J} \end{aligned}$$

where we remove the index over scale *j* by concatenating scales, hence $k = 1, 2, \ldots n*J$, where $J=\lfloor \log _2(n)\rfloor$. Similarly we define the spectra for each simulated series:

$$\begin{aligned} \varvec{S}^g_m = \left\{ S_{k,m}^g \right\} _{k = 1, 2, \ldots n*J}. \end{aligned}$$

To obtain a group spectra, an average is then taken over the M simulated series at each position of each scale for each group,

$$\begin{aligned} \varvec{\bar{S}}^g = \left\{ \frac{1}{M}\sum _{m=1}^M S_{k,m}^g \right\} _{k = 1, 2, \ldots n*J}. \end{aligned}$$

Based on these spectra, the distance metric proposed is a variance- corrected squared distance, across all spectral coefficients as proposed in Krzemieniewska et al. (2014),

3 $$\begin{aligned} D^g = \frac{M}{(M+1)} \sum _{k=1}^{n*J} \frac{(S^o_k - \bar{S}^g_k)^2}{\sum _{m=1}^{M} (S^{g}_{k,m} - \bar{S}^g_k)^2} \end{aligned}$$

Note that the variance correction occurs within the denominator to account for potentially different variability seen across simulations for each group. This is modified from Krzemieniewska et al. (2014) to allow different variances within each group. The theoretical consistency of the classification was shown in Theorem 3.1 from Fryzlewicz and Ombao (2009) where the error for misclassifying two spectra $\left\{ S_k^{(1)}\right\} _k$ and $\left\{ S_k^{(2)}\right\} _k$ (whose difference summed over *k* is larger than *CN*) is bounded by $\mathcal {O}\left( N^{-1}\log ^3_2{N} + N^{1/\{2\log _2(a)-1\}-1}\log ^2_2{N}\right)$. However, this result requires a short memory assumption that is clearly not satisfied for our long memory processes. Thus, we prove a similar bound under the assumption that the spectra are created from ARFIMA processes. We first replicate the required assumptions from Fryzlewicz and Ombao (2009) for completeness:

#### Assumption 2.1

(*Assumption 2.1 from* Fryzlewicz and Ombao (2009)) The set of those locations *z* where (possibly infinitely many) functions $S_j (z)$ contain a jump is finite. In other words, let $\mathcal {B} := \left\{ z : \exists j \lim _{u \rightarrow z^{-}} S_j (u) \ne \exists j \lim _{u \rightarrow z^{+}} \right\}$. We assume $B := \# \mathcal {B} < \infty$.

#### Assumption 2.2

(*Assumption 2.2 from* Fryzlewicz and Ombao (2009)) There exists a positive constant $C_1$ such that for all $j, S_j(z) \le C_1 2^j$.

#### Theorem 1

Suppose that assumptions 2.1 and 2.2 hold, and that the constants $\mathcal {P}_j$ from definition 1 decay as $\mathcal {O}(a^j)$ for $a>2$. Let $S_j^{(1)} (z)$ and $S_j^{(2)} (z)$ be two non-identical wavelet spectra from ARFIMA processes. Let $I^{(J)}_{k,N}$ be the wavelet periodogram constructed from a process with spectrum $S^{(1)} (z)$, and let $L_{k,N}^{(j)}$ be the corresponding bias-corrected periodogram, with $J^* = \log _2 N$. Let

$$\begin{aligned} \sum _{j,k} \left\{ S_j^{(1)} (k/N) - S_{j}^{(2)} (k/N) \right\} ^2 = \mathcal {O} (N). \end{aligned}$$

The probability of misclassifying $L_{k,N}^{(j)}$ as coming from a process with spectrum $S_j^{(2)} (z)$ can be bounded as follows:

$$\begin{aligned} P(D_1 > D_2) =\mathcal {O} \left( \log _2^2 N \left[ N^{-1} + N^{\frac{1}{(2\log _2 a - 1)}-1} \right] \right) \end{aligned}$$

#### Proof

The proof is given in Appendix 1.

A summary of the proposed procedure is given in Algorithm 1.

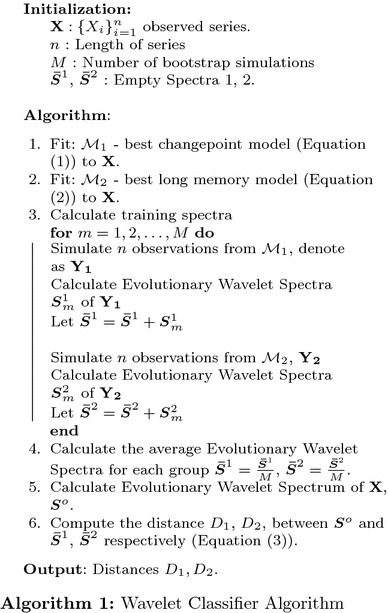



## Simulation study

To test the empirical accuracy of our proposed approach, simulations were conducted over a number of models. Here, these models are chosen over a number of parameter magnitudes and combinations to show the effectiveness of the approach outlined in Sect.  2. A number of these models also appear in Yau and Davis (2012) which uses a likelihood ratio method to test the null hypothesis of a changepoint model. Their results for these models are correspondingly given as a comparison.

**Table 1:** Changepoint observations results with likelihood ratio comparison (Yau and Davis 2012)

Ref	Model parameters						Classification rate			Y and D Likelihood ratio	
	$\lambda$	$\mu$	$\phi _1$	$\theta _1$	$\phi _2$	$\theta _2$	$n=512$	$n=1024$	$n=2048$	$n=500$	$n=1000$
1	0.5	1	0.1	0.3	0.4	0.2	1.00	1.00	1.00	0.99	0.97
2	0.5	2	0.1	0.3	0.4	0.2	1.00	1.00	1.00	0.95	0.93
3	0.5	1	0.1	0.3	0.8	0.2	1.00	1.00	1.00	0.97	0.99
4	0.5	2	0.1	0.3	0.8	0.2	1.00	1.00	1.00	0.94	0.95
5	0.7	1	0.1	0.3	0.8	0.2	1.00	1.00	1.00	0.94	0.94
6	0.7	2	0.1	0.3	0.8	0.2	1.00	1.00	1.00	0.91	0.93

For each model given in the tables below, 500 realisations of each model were generated and classified, using $M=1000$ training simulations for each fit. For computational efficiency, the maximum order of the fitted models is constrained to $p, q \le 1$. Three different time series lengths were computed for each model; 512, 1024 and 2048. It is expected that as a series grows larger, more evidence of long memory features will become prevalent, and as such the effect of length of series on accuracy is investigated.

We have used $n=2^J$ as the length of the series as the wavelet decomposition software (Nason 2016b) requires that the series transformed is of dyadic length. This is not a desirable trait as data sets come in many different sizes. Thus, we overcome this using a standard padding technique (Nason 2010) that adds 0’s to the left of each series until the data are of length $2^J$. The extended wavelet coefficients are then removed before calculating the distance metric.

### Changepoint observations

For the changepoint models, we used the simulations given in Yau and Davis (2012). Table 1 gives the parameters used in Eq. (1) along with the correct classification rate. The results show that if the data follow a changepoint model then we have a 100% classification rate. A movement of the changepoint to a later part of the series, as in models 5 and 6, does not appear to have an effect upon classification rates unlike for the Yau and Davis method. It is not really a surprise that we are receiving 100% classification rates as if a changepoint occurs then it is a clear feature within the spectrum.

It should be noted that as the Yau and Davis method is a hypothesis test we would expect results around 0.95 for a 5% type I error.

**Table 2:** Long memory observations result with likelihood ratio comparison (Yau and Davis 2012)

Ref	Model parameters				Classification rate			Y and D LR power
	$\phi$	*d*	$\theta _1$	$\theta _2$	$n=512$	$n=1024$	$n=2048$	$n=500$
7	$-$0.8	0.1	0.6		0.42	0.61	0.79	0.63
8	$-$0.8	0.2	0.6		0.56	0.83	0.94	0.97
9	$-$0.8	0.3	0.6		0.66	0.90	0.96	0.98
10	$-$0.8	0.4	0.6		0.75	0.88	0.96	0.96
11	0.1	0.1	$-$0.8		0.74	0.87	0.95	0.08
12	0.1	0.2	$-$0.8		0.84	0.96	0.99	0.09
13	0.1	0.3	$-$0.8		0.89	0.98	1.00	0.15
14	0.1	0.4	$-$0.8		0.88	0.99	1.00	0.32
15	0.1	0.1	0.8		0.54	0.78	0.90	
16	0.1	0.2	0.8		0.61	0.85	0.91	
17	0.1	0.3	0.8		0.62	0.87	0.95	
18	0.1	0.4	0.8		0.63	0.87	0.98	
19	0.6	0.1	$-$0.8		0.33	0.45	0.65	
20	0.6	0.2	$-$0.8		0.38	0.62	0.83	
21	0.6	0.3	$-$0.8		0.44	0.63	0.87	
22	0.6	0.4	$-$0.8		0.39	0.59	0.86	
23	0.0	0.1	0.7	$-$0.7	0.94	0.97	0.99	
24	0.0	0.2	0.7	$-$0.7	1.00	0.99	1.00	
25	0.0	0.3	0.7	$-$0.7	1.00	1.00	1.00	
26	0.0	0.4	0.7	$-$0.7	1.00	0.99	1.00	

**Fig. 2:**
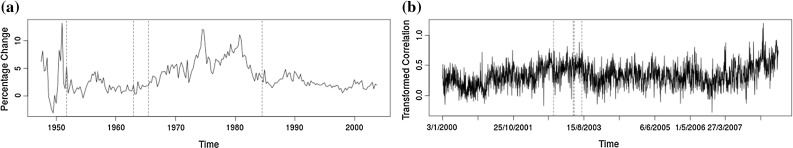
Real data examples. **a** Time series of US price inflation. **b** Time series of the cross-correlations of American Express and Home Depot.

### Long memory observations

In contrast to the changepoint models, the classification of a long memory model is expected to be less clear. This is due to the variation within the wavelet spectrum of long memory series that could be interpreted as different levels and hence a changepoint model would be more appropriate. To demonstrate the effect of the classifier on long memory observations, a larger number of models were considered. We simulated long memory models with differing levels of long memory as measured by the *d* parameter, values close to 0 are closer to short memory models and values close to 0.5 are stronger long memory models (values >0.5 are not stationary and thus not considered).

The results in Table 2 give an indication of the accuracy of the classifier in a number of different situations. Overall, as the length of the time series increases we see an increase in classification accuracy. This is to be expected as evidence of long memory will be more prevalent in longer series. Similarly as we increase the long memory parameter *d* from 0.1 to 0.4 we improve the classification rate.

Some interesting things to note include, when there are strong AR parameters ($\phi$) such as models 7–10 and 19–22 we require longer time series to achieve good classification rates. However, in contrast if there are strong MA components as in the remaining models the classifier performs better. A larger effect is found when the MA parameter is negative, seen through models 11–14 where the classifier performs strongly even at $n=512$. This effect is further exemplified by models 23–26 which include a further MA parameter and achieve near 100% classification at $n=512$. Here the maximum used *p*, *q* was 2.

Comparing our results to that of Yau and Davis we note that the opposite performance is seen. For the likelihood ratio method there is high power for models with strong AR components and poor performance for strong MA components. Notably the strong MA performance is much worse than our method on the strong AR components.

## Application

To further demonstrate the usage of our approach, two applications to real data are given in this section. The first is an economics example based on US price inflation and this is followed by financial data on stock cross-correlations. A sensitivity analysis was conducted over the possible maximum values of *p*, *q*. It was found that no additional parameters were required beyond maximum $p,q=4$, thus these results are presented here.

### Price inflation

US price inflation can be determined using the GDP index. The data set used here is available from the Bureau of Economic Analysis, based on quarterly GDP indexes, denoted $P_t$, from the first quarter of 1947 to the third quarter of 2006 (227 data points). Price inflation is calculated as $\pi _t = 400 \ln (P_t / P_{t-1})$ (thus $n=226$). A plot of the inflation is given below in Fig. 2a. Studies of the persistence of this data have been conducted to determine the level of dependence within the series. A high amount of persistence, indicating long memory, was found in Pivetta and Reis (2007). However Levin and Piger (2004) found a structural break, which when accounted for showed the series to have low persistence, indicating the presence of changepoints with short memory segments. Applying our classification approach to this series will give an additional indication as to which model is statistically more appropriate.

**Fig. 3:**
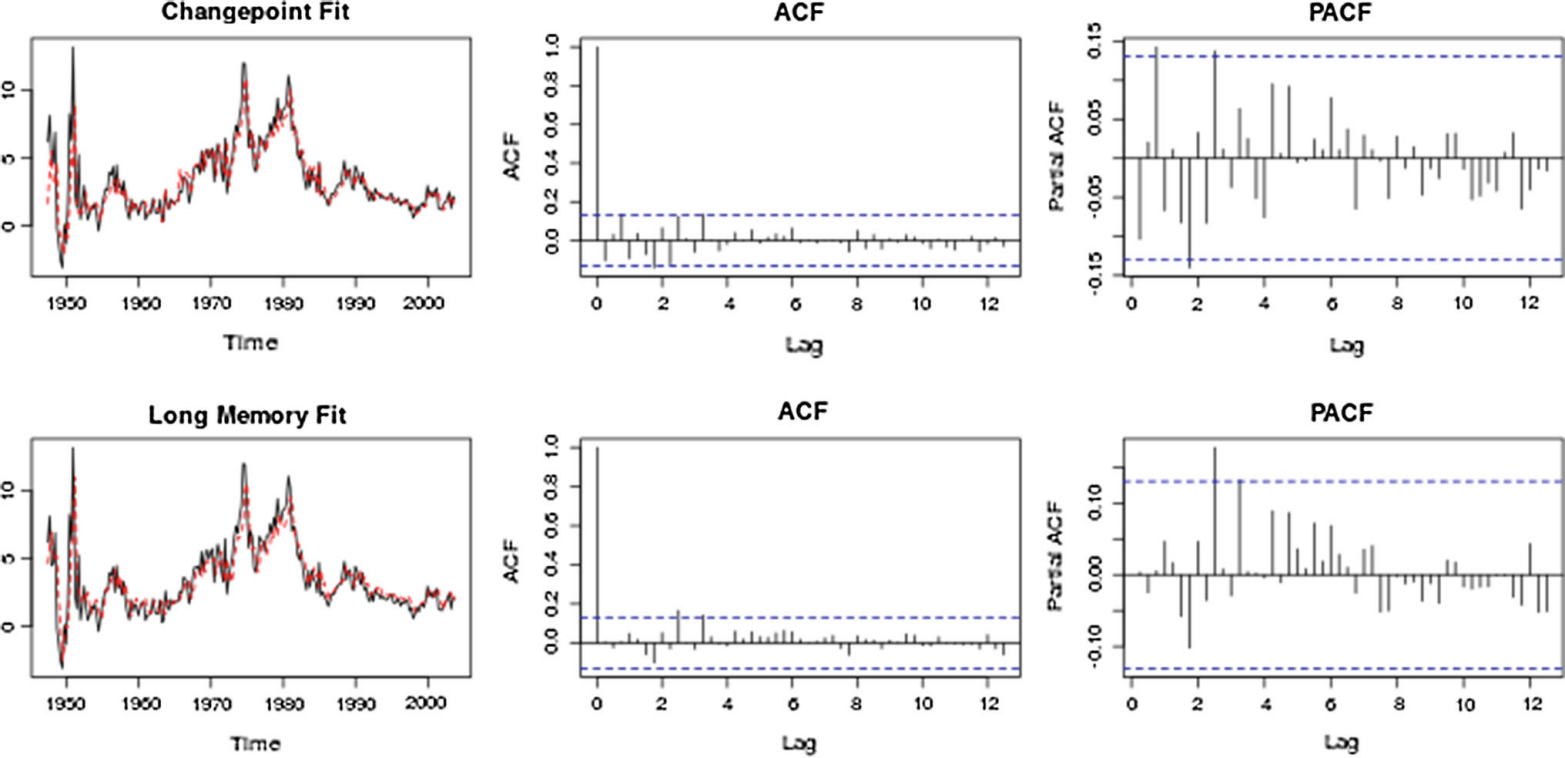
Inflation diagnostics. (*Top*) *Left* Original data with fitted changepoint model; *Middle* Autocorrelation function of changepoint model residuals; *Right* Partial autocorrelations of changepoint model residuals. (*Bottom*) *Left* Original data with fitted long memory model; *Middle* Autocorrelation function of long memory model residuals; *Right* Partial autocorrelations of long memory model residuals

The parameters of the fitted changepoint and long memory models are given in Table 3. Diagnostic autocorrelation and partial autocorrelation function plots are given in Fig. 3. The level shifts are given in respect to their position in the series, but correspond to 1951 Q3, 1962 Q4, 1965 Q2, 1984 Q2. The classifier returns a changepoint classification for this series.

**Table 3:** Model fits and scores for US inflation (inflation) and stock cross-correlations (stock)

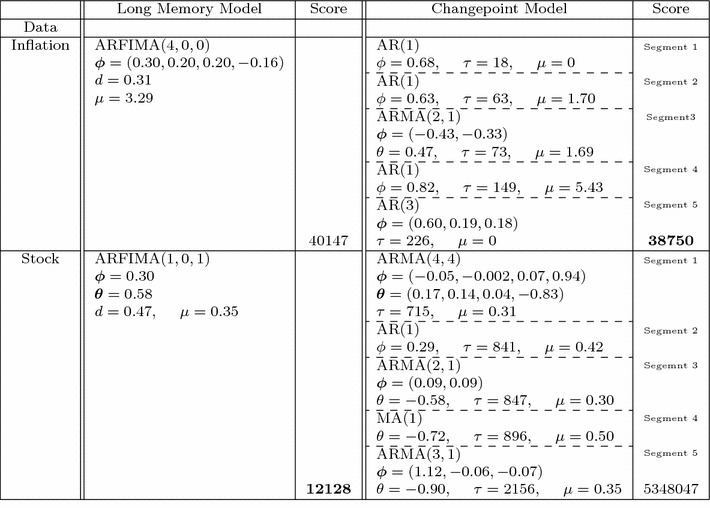	

Bold scores are the minimum. Each segment ending at $\tau$ is separated by a dotted line

**Fig. 4:**
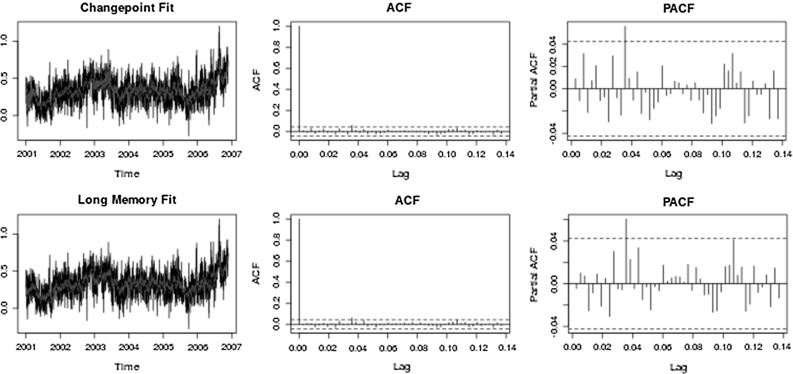
Stock diagnostics. (*Top*) *Left* Original data with fitted changepoint model; *Middle* Autocorrelation function of changepoint model residuals; *Right* Partial autocorrelations of changepoint model residuals. (*Bottom*) *Left* Original data with fitted long memory model; *Middle* Autocorrelation function of long memory model residuals; *Right* Partial autocorrelations of long memory model residuals

### Stock cross-correlations

Stock cross-correlation data have been obtained from the supplementary material of Chiriac and Voev (2011). The data consist of open to close stock returns for 6 companies from January 1st 2001 to 30th July 2008 ($n=2156$). The data are first transformed using a Fisher transformation, then correlations are calculated between each stock. Here analysis will look at the correlation between American Express and Home Depot.

These data have been analysed previously by Bertram et al. (2013) to determine between fractional integration (long memory behaviour) and level shifts and are given in Fig. 2b. Parameters for the models fitted by the algorithm are also in Table 3. It can be seen that one of the AR coefficients is close to 1 indicating an element of non-stationarity; however, we conducted a test of stationarity on this segment using the locits R package (Nason 2016a) which implements the test of stationarity from Nason (2013) (no rejections) and also the fractal R package (Constantine and Percival 2016) which implements the Priestley–Subba Rao (PSR) test (Priestley and Rao 1969) (time- varying *p* value 0.061). This coupled with autocorrelation and partial autocorrelation function plots given in Fig. 4 means we conclude that the segment is stationary. Here the estimated changepoints at times 715, 841, 847 and 896 correspond 15/12/2002, 20/04/2003, 26/04/2003 and 14/06/2003. The distance scores given by the classifier indicate a strong preference for long memory over changepoints. This result stands against that found in Bertram et al. (2013) which indicated a preference for a model with similarly 4 changepoints. The difference is likely due to the fact that in Bertram et al. (2013) the changepoint model does not contain any short memory dependence and we have shown here that if that short memory structure is correctly taken into account within the sub-series then the series shows greater evidence of long memory properties.

## Conclusion

The wavelet classification process presented within this paper provides the user a distinct choice over a number of proposed models, and when explicitly applied to an ambiguity such as long memory or a changepoint as in Sect. 3, it provides an additional piece of information to aid decision-making. The accuracy of the classifier over a number of simulated models has been presented within Sect. 3 and applied to data from the financial and economic fields in Sect.  4.

The evolutionary wavelet spectrum provides a representation of non-stationarity which is lacking in the commonly used (averaged over time) spectrum. This gives an advantage when drawing comparisons between non-stationary and stationary series, since the wavelet spectrum may appear substantially different. Quantifying this visual difference allows for a direct comparison between the series and each proposed model.

The variance-corrected squared distance metric used in the proposed classifier has been demonstrated to be quite accurate under the ambiguity of long memory and changepoint models. It is particularly effective at identifying changepoint models correctly, as the results in Table 1 demonstrate. It was noted that there is relatively lower variation between the simulations generated for the changepoint than the long memory model, which reduces the distance metric significantly even though it is variance corrected.

As mentioned in Sect. 1 there are many series that can be found in fields such as economics and finance which show evidence of the ambiguity investigated here. This classification is not intended to propose a final model for these series, but instead give additional information, treated perhaps as a diagnostic. This could be to begin investigation of a series, or to confirm a previously found model fit. As this is not a formal test, the lack of assumptions allows for more flexibility in how the classification can be used. This work, however, is not restricted only to the ambiguity mentioned here, further work could extend it to determine between other features, such as local trends and seasonal behaviour or combining the behaviour of both models, i.e. a long memory model with a changepoint.

An aspect not covered in this paper is the precise form of ARMA and long memory models in the LSW paradigm, i.e. how the model coefficients relate to the $W_{j,k}$’s. This is an interesting area for future research which would cement the LSW model as an encompassing model but is beyond the scope of this paper.

An R package (LSWclassify) is available from the authors that implements the method from the paper.

## Appendix: Proof of Theorem 1

### Proof

We replicate the steps of the proof within the Appendix of Fryzlewicz and Ombao (2009) up until (A.6), where following this step the short memory condition is used. To briefly summarise previous steps,

$$\begin{aligned} P(D_1 - D_2> 0)&= P(X - t > 0) \le E( \tilde{X}^2)/t^2, \\&\quad {\text {(by Chebyshev's Inequality)}} \\ E(\tilde{X}^2)&=: I + II, \\ I&\le C J_0 J^* \sum _{j=-1}^{-J0} \sum _{i =-1}^{-J^*} 2^{i+j} E \left\{ b_{i,j}^{2} \right\} , \\ E \left\{ b_{i,j}^{2} \right\}&=: 2A + 2B. \end{aligned}$$

Definitions for these components can be found in the original proof. Component A is where we alter the proof.

Recall $I_{k,N}^{(i)}$ is the wavelet periodogram at a fixed scale *i*, at position *k* with total length *N*, with $d_{k,N}^{(i)}$ the wavelet coefficient corresponding to it through the relationship $I_{k,N}^{(i)} = (d_{k,N}^{(i)})^2$. We continue the proof from (A.6) using the ARFIMA assumption instead. Following from above (A.6):

4 $$\begin{aligned} A&= E \left\{ \sum _{k=1}^{N} \left\{ I_{k,N}^{(i)} - E \left( I_{k,N}^{(i)} \right) \right\} c_{j,k} \right\} ^2 \nonumber \\&\le 2^{2j} \sum _{k,k^{\prime }=1}^{N} \left| \text {cov} \left( I_{k,N}^{(i)}, I_{k^{\prime },N}^{(i)} \right) \right| \nonumber \\&= 2^{2j} \sum _{k,k^{\prime }=1}^{N} 2 \text {cov}^2\left( d_{k,N}^{(i)}, d_{k^{\prime },N}^{(i)} \right) \nonumber \\&\quad (\text {by Isserli's Theorem)} \end{aligned}$$

Jensen (2000) gives bounds for the covariance of wavelet coefficients;

$$\begin{aligned} \text {cov}\left( d_{k,N}^{(m)}, d_{n,N}^{(j)} \right)&= \text {C}_1 | \alpha |^{2d-1-2M} + R_{2M+1} \\ \alpha&= 2^{m-j} k - n, \quad m \ge j \\ |R_{2M+1}|&\le \text {C}_2 |\alpha |^{2d-2-2M}, \end{aligned}$$

where $M \ge 1$ is the number of vanishing moments in the wavelet used. Using $|\alpha | = |2^{i-i}k - k^{\prime }| = |k-k^{\prime }| \ge 1$ and substituting into Eq. (4):

$$\begin{aligned} A&= 2^{2j+1} \sum _{k,k^{\prime }=1}^{N} (\text {C}_3 |\alpha |^{2d-1-2M} + R_{2M+1})^{2} \\&= 2^{2j+1} \sum _{k,k^{\prime }=1}^{N} |\text {C}_3 |\alpha |^{2d-1-2M} + R_{2M+1}|^{2} \\&\le 2^{2j+1} \sum _{k,k^{\prime }=1}^{N} ( \left| \text {C}_3 |\alpha |^{2d-1-2M}\right| + |R_{2M+1}|)^2 \\&\le 2^{2j+1} \sum _{k,k^{\prime }=1}^{N} ( \text {C}_4 |\alpha |^{2d-1-2M} + \text {C}_5|\alpha |^{2d-2-2M})^2 \end{aligned}$$

As $|\alpha |^{2d-2-2M} \le |\alpha |^{2d-1-2M}$ we have:

$$\begin{aligned} A&\le 2^{2j+1} \sum _{k,k^{\prime }=1}^{N} \left( \text {C}_6 |k - k^{\prime }|^{2d-1-2M} \right) ^2 \\&= 2^{2j+1} \sum _{k,k^{\prime }=1}^{N} \text {C}_7 \frac{1}{|k - k^{\prime }|^{-2(2d-1-2M)}} \\&= 2^{2j+1} \sum _{s=1}^{N-1} (N-s) \text {C}_7 \frac{1}{s^{-2(2d-1-2M)}} \\&= 2^{2j+1} \text {C}_7\left[ N \sum _{s=1}^{N-1} \frac{1}{s^{-2(2d-1-2M)}} - \sum _{s=1}^{N-1} \frac{1}{s^{-4(d-M) +1}} \right] \end{aligned}$$

Given that $|d| < 0.5$ and $M\ge 1$ then $4 < -2(2d-1-2M) = \delta _1$ and $3 < -4(d-M) + 1 = \delta _2$. We can then replace the sums using the definition of generalised harmonic numbers and their convergence:

$$\begin{aligned} H_{n,m}&= \sum _{k=1}^{n} \frac{1}{k^m} \\ H_{n,m}&= \mathcal {O}(1) \quad \text {as } n \rightarrow \infty {\quad } (m > 1) \end{aligned}$$

Thus

$$\begin{aligned} A&\le 2^{2j+1} \text {C}_7\left( N H_{N-1, \delta _1} - H_{N-1, \delta _2} \right)&= 2^{2j+1} C_7 \mathcal {H}_N, \end{aligned}$$

where $\mathcal {H}_N=N H_{N-1, \delta _1} - H_{N-1, \delta _2}$. Returning to consider (A.4) from Fryzlewicz and Ombao (2009), we find a bound for component *I*, where $J_0, J^* = \log _2 N$ and $\varDelta = \frac{1}{(2\log _2 a - 1)}$:

$$\begin{aligned} I&= \text {C}_8 J_0 J^{*} \sum _{j=-1}^{-{J_0}} \sum _{i=-1}^{-{J^*}} 2^{i+j} \left[ 2^{2j+1} \text {C}_7 \left( N H_{N-1, \delta _1} \right. \right. \\&\quad \left. \left. - H_{N-1, \delta _2} \right) + N^{1 + \varDelta } 2^j \right] \\&= \text {C}_8 \log _2^2 N \sum _{j=-1}^{-{J_0}} 2^{j} \left[ 2^{2j+1} \text {C}_7 \mathcal {H}_N \right. \\&\quad \left. +\, N^{1 + \varDelta } 2^j \right] \left( 1-2^{J*} \right) \\&= \text {C}_8 \log _2^2 N \left[ \sum _{j=-1}^{-{J_0}} \text {C}_7 2^{3j+1} \left( 1-2^{J*} \right) \mathcal {H}_N \right. \\&\quad \left. + \sum _{j=-1}^{-{J_0}} 2^{3j} \left( 1-2^{J*} \right) N^{1 + \varDelta } \right] \\&= \text {C}_9 \log _2^2 N \left( 1-2^{-J*} \right) \mathcal {H}_N \sum _{j=-1}^{-{J_0}} 2^{3j+1} \\&\quad + \text {C}_8 \log _2^2 N \left( 1-2^{-J*} \right) N^{1 + \varDelta } \sum _{j=-1}^{-{J_0}} 2^{3j} \\&= \text {C}_9 \log _2^2 N \left( 1-2^{-J*} \right) \mathcal {H}_N \frac{2}{7} \left( 1 - 2^{-3{J_0}} \right) \\&\quad + \text {C}_8 \log _2^2 N \left( 1-2^{-J*} \right) N^{1 + \varDelta } \frac{1}{7} \left( 1 - 2^{-3{J_0}} \right) \\&= \log _2^2 N \left( 1-N^{-1} \right) \left( 1 - N^{-3} \right) \\&\quad \times \left[ \text {C}_{10} \mathcal {H}_N + \text {C}_{11} N^{1 + \varDelta } \right] \\&= \log _2^2 N \left( 1 - N^{-3} - N^{-1} + N^{-4} \right) \\&\quad \times \left[ \text {C}_{10} \mathcal {H}_N + \text {C}_{11} N^{1 + \varDelta } \right] \\&\le \text {C}_{12} \log _2^2 N \left( \mathcal {H}_N + N^{1 + \varDelta } \right) \end{aligned}$$

Following this, using results in Fryzlewicz and Ombao (2009) the probability of misclassification is:

$$\begin{aligned} P(X > t) = \mathcal {O} \left( \log _2^2 N \left[ N^{-1} + N^{\varDelta -1} \right] \right) . \end{aligned}$$
